# Isotopic niche overlap between sympatric Australian snubfin and humpback dolphins

**DOI:** 10.1002/ece3.8937

**Published:** 2022-05-24

**Authors:** Guido J. Parra, Zachary Wojtkowiak, Katharina J. Peters, Daniele Cagnazzi

**Affiliations:** ^1^ Cetacean Ecology, Behavior and Evolution Lab College of Science and Engineering Flinders University Adelaide South Australia Australia; ^2^ Evolutionary Genetics Group Department of Anthropology University of Zurich Zurich Switzerland; ^3^ School of Earth and Environment University of Canterbury Christchurch New Zealand; ^4^ 4571 Marine Ecology Research Centre Faculty of Science and Engineering Southern Cross University Lismore New South Wales Australia

**Keywords:** cetaceans, ecological niche, feeding ecology, *Orcaella heinsohni*, resource partitioning, SIBER, *Sousa sahulensis*, stable isotopes

## Abstract

Ecological niche theory predicts the coexistence of closely related species is promoted by resource partitioning in space and time. Australian snubfin (*Orcaella heinsohni*) and humpback (*Sousa sahulensis*) dolphins live in sympatry throughout most of their range in northern Australian waters. We compared stable isotope ratios of carbon (δ^13^C) and nitrogen (δ^15^N) in their skin to investigate resource partitioning between these ecologically similar species. Skin samples were collected from live Australian snubfin (*n* = 31) and humpback dolphins (*n* = 23) along the east coast of Queensland in 2014–2015. Both species had similar δ^13^C and δ^15^N values and high (>50%) isotopic niche space overlap, suggesting that they feed at similar trophic levels, have substantial dietary overlap, and rely on similar basal food resources. Despite similarities, snubfin dolphins were more likely to have a larger δ^15^N value than humpback dolphins, indicating they may forage on a wider diversity of prey. Humpback dolphins were more likely to have a larger δ^13^C range suggesting they may forage on a wider range of habitats. Overall, results suggest that subtle differences in habitat use and prey selection are likely the principal resource partitioning mechanisms enabling the coexistence of Australian snubfin and humpback dolphins.

## Graphical abstract

We compared stable isotope ratios of carbon (δ^13^C) and nitrogen (δ^15^N) in the skin of Australian snubfin and humpback dolphins to investigate resource partitioning between these ecologically similar species. Both species had similar δ^13^C and δ^15^N values and high (>50%) isotopic niche space overlap, suggesting that they feed at similar trophic levels, have substantial dietary overlap, and both rely on similar basal food resources. Despite similarities, subtle differences in δ^13^C and δ^15^N suggest differences in habitat use and prey selection are likely the principal resource partitioning mechanisms enabling their coexistence.

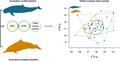

## 1 INTRODUCTION

Natural ecosystems are composed of assemblages of coexisting species occupying a variety of trophic levels. The ecological interactions (e.g., competition, predation) occurring between these coexisting species have a strong influence on the structure and function of animal communities, and hence promote biodiversity (Chesson, 2000; Tokeshi, 1999). Upper trophic level consumers can play important ecological roles in the maintenance of marine, terrestrial, and freshwater ecosystem function and health through consumptive (predation) and non‐consumptive (predatory and non‐predatory risk) effects (Estes et al., 2011). Therefore, identifying the mechanisms that promote coexistence and the trophic relationships among high‐trophic level predators is critical to understand community structure and dynamics, and for ecosystem management and conservation.

Sympatric species with similar ecological requirements may compete for limited resources, which may lead to the exclusion of the less competitive species (Roughgarden, 1983). Coexisting species are thus expected to differ in ecological requirements to minimize niche overlap and avoid interspecific competition (Chesson, 2000; MacArthur & Levins, 1967). Niche differentiation can promote species coexistence; therefore, quantifying the degree of niche overlap among co‐occurring species is an important tool for gaining insights into how closely related species coexist and the functional position and role they play within their environment (Broennimann et al., 2012; Geange et al., 2011; Lu et al., 1989).

Several species of delphinids coexist in sympatry (Bearzi, 2005; Parra, 2006; Syme et al., 2021). Delphinids eat a wide variety of prey, including fish, cephalopods, crustaceans, and even other marine mammals, and thus can act as apex‐ or mesopredators within marine and freshwater ecosystems (Kiszka et al., 2015). Given their high trophic position and relatively large body size, they can consume substantial amounts of prey biomass (Bearzi et al., 2010; Williams et al., 2004) and can have important effects on the overall trophic dynamics of marine ecosystems through direct predation and risk effects (Estes et al., 2016; Kiszka et al., 2015). However, relatively little is known about the ecological role of these dolphin communities, how they coexist, and their influences on the structure and function of marine ecosystems.

Australian snubfin (*Orcaella heinsohni*) and humpback dolphins (*Sousa sahulensis*), hereafter referred to as ‘snubfin dolphin’ and ‘humpback dolphin’, respectively, are primarily found in shallow (<30 m deep) tropical/subtropical coastal waters of the Sahul Shelf from the southern waters of New Guinea to, and across, northern Australian waters (Beasley et al., 2005; Jefferson & Rosenbaum, 2014). Both species co‐occur throughout most of their range in northern Australian waters and are known to live in direct sympatry across several locations (Parra et al., 2002, 2004). Ecologically, both species are relatively similar: both occur in small populations of typically fewer than 150 individuals, show a high degree of overlap in space use, and have similar patterns of habitat use and behavioral activities according to space and time, to the point that both species are recorded frequently in mixed species groups (Parra, 2005, 2006; Parra et al., 2006, 2006, 2011). Thus, segregation into exclusive ranges in space and time, and difference in habitat use and behavior patterns, do not seem to fully explain their coexistence.

Dietary niche partitioning is the primary way many carnivore species limit interspecific competition (Donadio & Buskirk, 2006) and a key mechanism regulating coexistence in marine mammals (Durante et al., 2021; Gibbs et al., 2011; Giménez et al., 2018). Slight differences in habitat preferences and diet appear to be some of the principal factors promoting the coexistence of snubfin and humpback dolphins (Parra, 2006; Parra & Jedensjö, 2014). Previous studies have shown that snubfin dolphins in northern Queensland preferred slightly shallower (1–2 m) waters than humpback dolphins (2–5 m), and favored seagrass beds more often than did humpback dolphins (Parra, 2006). Stomach content analysis showed the diet of snubfin and humpback dolphins overlapped partially, particularly across the fish taxa consumed by both species (Parra & Jedensjö, 2014). However, humpback dolphins appeared to favor fish, while snubfin dolphin diet included a large amount of cephalopods (Parra & Jedensjö, 2014).

While stomach content analysis is a valuable tool in studies of diet composition, it can be biased due to inherent problems in the sampling regime and prey identification (Pierce & Boyle, 1991; Santos et al., 2001). Stomach contents can only be collected from dead stranded animals, which limits sampling opportunities (Barros et al., 2004; Matley et al., 2015), and stranded animals may have been engaged in abnormal feeding behavior before stranding due to illness (Owen et al., 2011); misrepresenting the actual diet of a healthy animal. Stomach contents are also biased toward hard parts such as otoliths and beaks, which are resistant to digestion, and may cause overestimation of the importance of particular prey such as cephalopods (Bowen & Iverson, 2013). In addition, erosion of hard parts may result in misidentification of prey species (Dunshea et al., 2013). These limitations prevent a clear understanding of dietary partitioning between sympatric species based on stomach content analyses alone.

Comparisons of carbon δ^13^C and nitrogen δ^15^N values among consumers (i.e., the isotopic niche) can provide a quantitative indication of an organism’s trophic niche (Marshall et al., 2019; Newsome et al., 2007). Carbon isotope ratios can differ in a marine system due to temperature differences, surface‐water CO_2_ concentrations and differences in plankton biosynthesis or metabolism, and thus indicate likely carbon sources relating to feeding habitat (Ben‐David & Flaherty, 2012; Kelly, 2000; Rubenstein & Hobson, 2004). Nitrogen isotope ratios can be used to estimate the consumer’s trophic position given the well‐established stepwise enrichment (3–4‰) of ^15^N in the body tissue of organisms with increasing trophic level (Minagawa & Wada, 1984; Post, 2002).

To better understand the feeding ecology of snubfin and humpback dolphins, we investigated differences in their δ^13^C and δ^15^N values to assess isotopic niche width and overlap of niche space. We hypothesized that snubfin and humpback dolphins (1) have similar foraging habitats and trophic levels and that this would be reflected in comparable δ^13^C and δ^15^N values, (2) have similar trophic niches that would be reflected by a high degree of overlap of their isotopic niche spaces, and that (3) snubfin dolphins would have greater δ^15^N ranges, given they feed on a wider diversity of prey (fish and cephalopods) than humpback dolphins.

## 2 METHODS

### 2.1 Study area and sample collection

**FIGURE 1:**
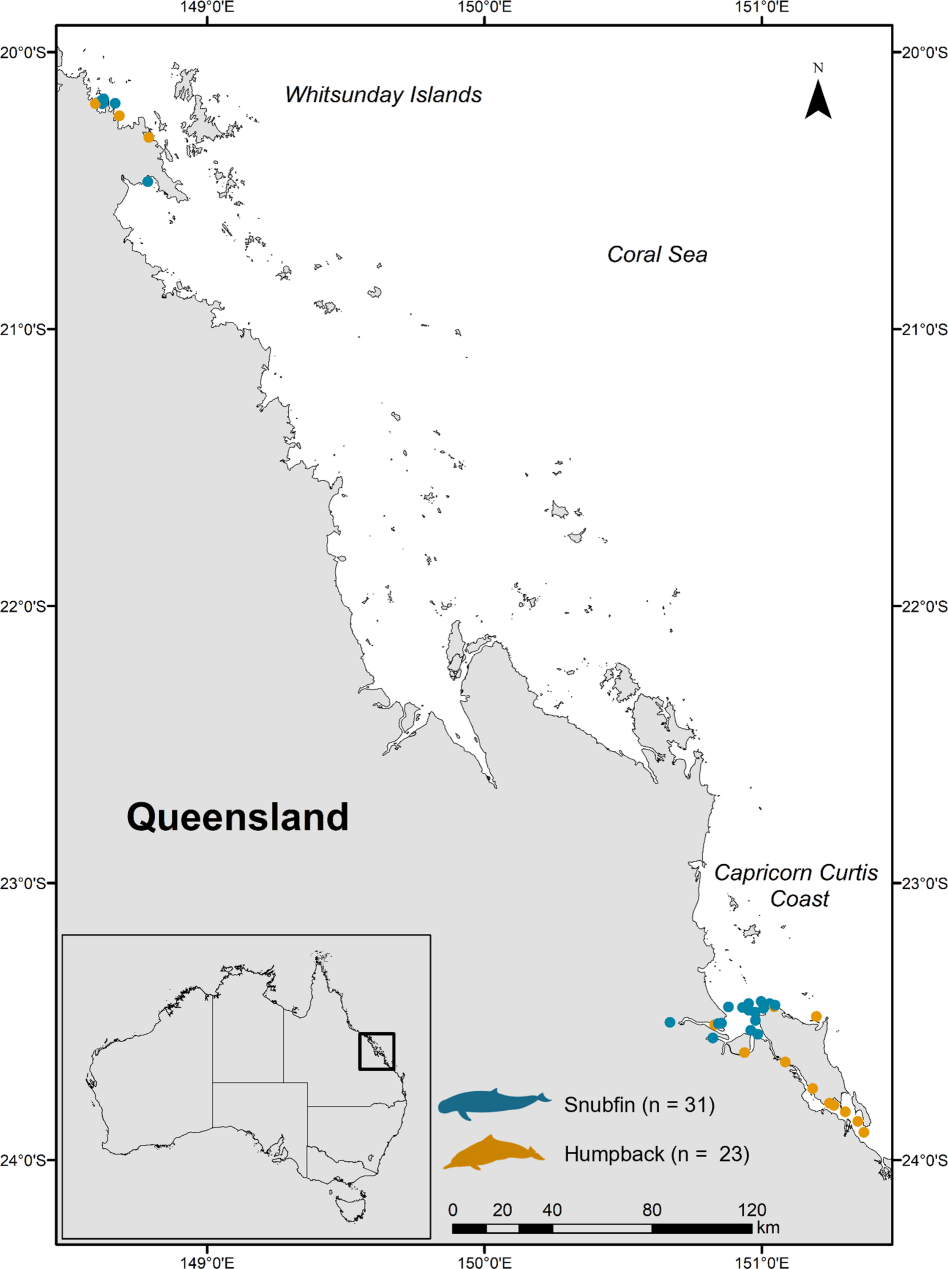
Map showing the locations of Australian snubfin and humpback dolphins biopsy sampled along coastal waters off the eastern coast of Queensland, Australia

We collected skin samples of adult snubfin (*n* = 31) and humpback dolphins (*n* = 23) using a PAXARMS biopsy rifle (Krützen et al., 2002) during boat‐based surveys in coastal waters of the Whitsundays and Capricorn‐Curtis Coast region, east coast of Queensland (Figure 1) between January 2014 and September 2015. Photos of each individual’s dorsal fin were taken at the time of biopsy sampling for photo identification and to prevent re‐sampling of individuals. Skin samples were transferred into liquid nitrogen prior to being stored at −80°C until stable isotope analysis at the Centre for Coastal Biogeochemistry Research, Southern Cross University.

### 2.2 Sample preparation and stable isotope analysis

Preparation of skin samples followed standard protocols for stable isotope analysis (Browning et al., 2014). Approximately 10 mg of skin was cut from each sample using a stainless‐steel scalpel sterilized with ethanol between cuts to prevent cross‐contamination of samples. The skin pieces were then transferred into Eppendorf capsules and oven‐dried at 60°C for 24 h to remove all moisture. Once dried, samples were ground into a fine powder using a mortar and pestle (which were sterilized with acetone between samples). Cetacean skin is known to have a high lipid content, which can lead to decreased δ^13^C values due to the ^12^C enrichment in the lipids (Giménez, Ramírez, et al., 2017; Lesage et al., 2010; Ryan et al., 2012). To minimize variance from lipid content all samples were lipid‐extracted by adding 5 ml of 2:1 chloroform‐methanol solution to the powdered samples, which were then vortexed for 30 s to ensure proper mixing (Post et al., 2007). Lipid‐extracted samples were then placed in a centrifuge for 5 min at 1000 rpm; the remaining solution was discarded and samples were again oven‐dried at 60°C for 24 h to remove residual solvent. Depending on the amount of sample available after processing, aliquots of 0.05 to 0.9 mg of powdered sample were sealed in tin capsules. Samples were measured using a Thermo Fisher DELTA V plus isotope ratio mass spectrometer (IRMS). The IRMS was coupled to an elemental analyzer (Thermo Fisher Flash EA) via an interface (Thermo Fisher Conflo IV). Samples were loaded into the elemental analyzer using an autosampler (Carvalho et al., 2020). Samples were measured along working standards (glycine: δ^13^C = −41.8, δ^15^N = 2.0; glucose: δ^13^C = −10.5; collagen: δ^13^C = −21.5, δ^15^N = 4.8). These working standards had previously been calibrated using international reference materials (USGS64: δ^13^C = −40.8, δ^15^N = 1.8; USGS65: δ^13^C = −20.3, δ^15^N = 20.7; USGS64: δ^13^C = −0.7, δ^15^N = 40.8) (Schimmelmann et al., 2016). Precision for δ^13^C was better than 0.15 ppt, and better than 0.3 ppt for δ^15^N, which is expected for this kind of analysis (Meier‐Augenstein, 2017).

Isotopic ratios were transformed into parts per thousand (‰) using delta notation (δ):

$$\delta X\left(‰\right)=\left(\left(\frac{R_{\mathrm{sample}}}{R_{\mathrm{standard}}}\right)‐1\right)\times 1000$$

where δX is δ^13^C or δ^15^N, *R*_sample_ is the ratio of light and heavier stable isotope in the sample, and *R*_standard_ is the ratio of stable isotopes in the standard reference materials.

### 2.3 Statistical analysis

We tested δ^15^N and δ^13^C data for each species for homogeneity of variance (non‐parametric Levene’s test) and normality (Shapiro‐Wilk’s test). Tests revealed homogeneity of variance for species, but assumptions of normality were not met for δ^13^C values for humpback dolphins (*p* = .04). We therefore used a one‐sided randomization test with 10,000 permutations at 0.05 significance level to investigate differences in isotopic values between species. This test compares the difference of the mean δ^15^N and δ^13^C values per species with the difference obtained by randomly allocating the observed isotopic values among the two species (Manly, 2007).

We used six metrics, proposed by Layman et al. (2007), to compare the isotopic niches of Australian snubfin and humpback dolphins:

1. δ^15^N range, which is the difference between the highest and lowest δ^15^N values of each species. δ^15^N range provides information on the range of trophic levels at which each species has been feeding.
2. δ^13^C range, as a measure of the difference between the highest and lowest δ^13^C values of each species. δ^13^C range provides an estimate of the variability of trophic sources of each species.
3. Total area (TA), which is a measure of the total amount of niche space occupied by a species in ‰^2^. TA was calculated from a convex hull drawn around the most extreme data points on an isotope δ^13^C–δ^15^N bi‐plot. As TA is sensitive to differences in sample size, because the area can only increase as new data points are added, we used the corrected version of the standard ellipse area (SEA_C_) as a measure of the mean core area (40%) of each species isotopic niche (Jackson et al., 2011).
4. Mean distance to centroid (CD) is the mean Euclidean distance of each individual of a population to the δ^15^N–δ^13^C centroid, where the centroid is the mean δ^15^N–δ^13^C value for all species in the food web. CD provides an estimate of overall dietary diversity.
5. Mean nearest neighbor distance (MNND) is the average nearest‐neighbor Euclidean distance between an isotopic coordinate relative to all other coordinates within a species. MNND provides an estimate of species packing and shows how similar or dissimilar the members of a population are to one another.
6. Standard deviation of nearest neighbor distance (SDNND). A measure of the evenness of spatial density and packing of individuals. Low SDNND values indicate a more even distribution of trophic niches.

We bootstrapped all Layman metrics with replacement (*n* = 10,000, indicated with a subscript “boot”) based on the smallest sample size in the data set (*n* = 23) to enable statistical comparison between dolphin species (Jackson et al., 2012; Manly, 2007). To further assess niche widths and isotopic niche overlap between species, we followed a Bayesian approach using multivariate ellipse‐based metrics (Jackson et al., 2011). This method is particularly useful when comparing groups with small sample sizes, as it corrects for the influence of outliers. We calculated standard ellipse areas (SEAs), which are the bivariate equivalent to standard deviation in univariate analyses. We also calculated SEA corrected (SEA_C_) to minimize bias introduced by small sample sizes. In addition, we calculated SEA_B_ (Bayesian SEA) using 1000 posterior draws to statistically compare niche width between species. We used SEA_B_ to calculate the niche overlap between Australian snubfin and humpback dolphins, calculated as the proportion of the total SEA_B_ for each species respectively. We calculated all metrics using the package SIBER (Stable Isotope Bayesian Ellipses in R) in R version 4.1.0 (Jackson et al., 2011; R Core Team, 2022).

## 3 RESULTS

**FIGURE 2:**
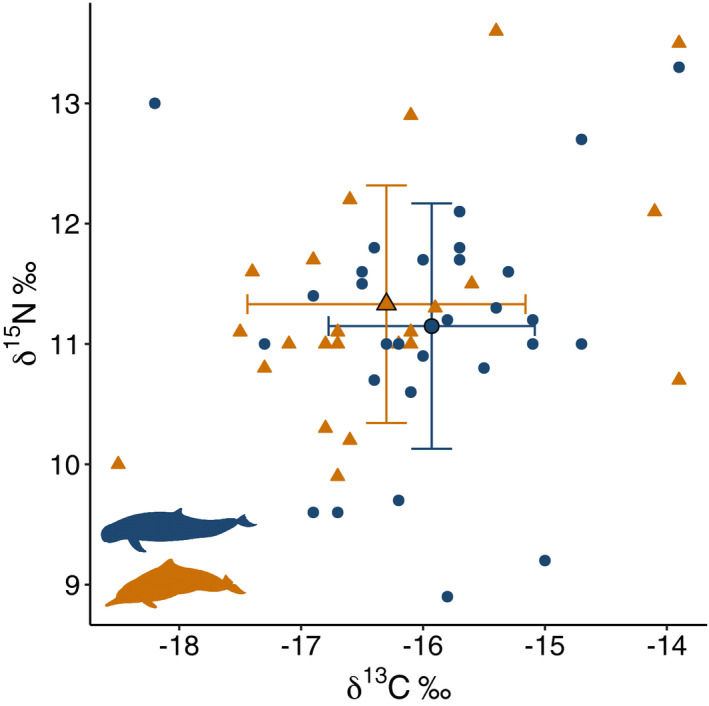
Isotopic values (δ^15^N and δ^13^C ‰) of Australian snubfin (blue circles) and humpback dolphins (gold triangles). Larger symbols with black outline and bars represent mean ±1 SD for each species, smaller symbols show individual values

Snubfin dolphins δ^13^C values ranged from −18.2 to −13.9‰ (mean ± SD = −15.93 ± 0.85‰), and δ^15^N values varied from 8.9 to 13.3‰ (mean ± SD =11.15 ± 1.02‰) (Figure 2). Humpback dolphins δ^13^C values varied from −18.5 to −13.9‰ (mean ± SD = −16.30 ± 1.14‰); and δ^15^N values varied from 9.9 to 13.6‰ (mean ± SD = 11.33 ± 0.99‰) (Figure 2). We found no difference between species in mean isotopic values for either δ^13^C or δ^15^N values (randomization test, *p* > .05).

**FIGURE 3:**
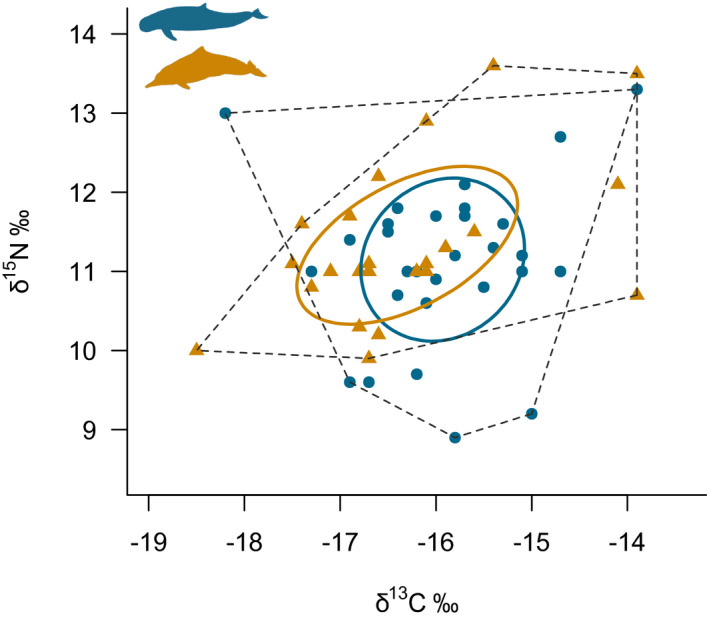
Total amount of niche space (TA, convex hull area‐dotted line) for Australian snubfin (blue circles) and humpback dolphins (gold triangles) and standard ellipse area corrected for small sample size (SEA_C_, solid lines). Ellipse areas hold 40% of the data

**FIGURE 4:**
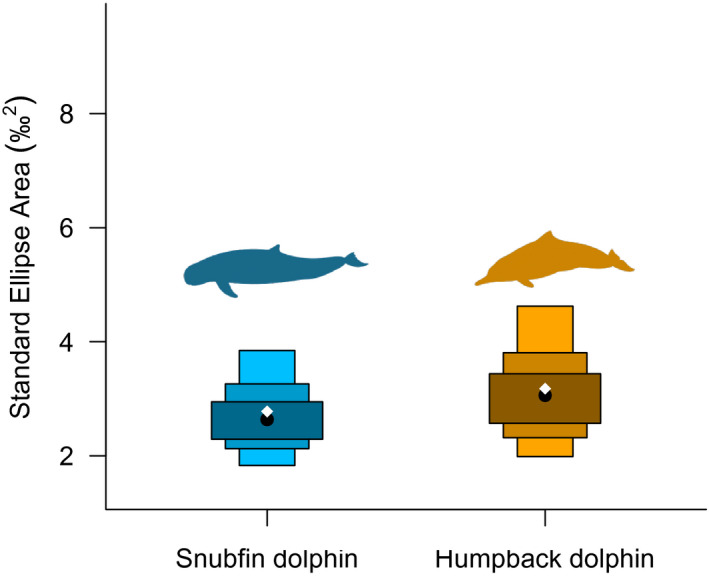
Density plot showing the 95, 75, and 50% credible intervals of standard ellipses area for Australian snubfin and humpback dolphins using Bayesian techniques. Black dots represent the mean standard ellipses area (SEA) for each species, white diamonds indicate the corrected standard ellipses area (SEA_C_)

Snubfin dolphins had a greater δ^15^N range than humpback dolphins (4.4‰ vs 3.7‰, respectively, bootstrapped probability of 80.1%) (Table 1). Values for δ^13^C range were similar across both dolphin species (snubfin: 4.3‰ and humpback: 4.6‰), with bootstrapping indicating humpback dolphins were slightly more likely (84.8%) to have a larger δ^13^C range than humpback dolphins (Table 1). The total amount of niche space (TA) was greater in snubfin dolphins (12.07‰^2^) than in humpback dolphins (10.66‰^2^); however, bootstrapping indicated the opposite with a 72.0% probability (Table 1, Figure 3). The mean core area (40%) of isotopic niche (SEA) was relatively similar for both species for all three types of ellipses (SEA_C_: snubfin: 2.78‰^2^ and humpback: 3.18‰^2^, see Table 1 for SEA and SEA_B_), although humpback dolphins were slightly more likely to have higher values than snubfin dolphins when comparing SEA_B_ (68.5% probability) (Table 1, Figure 4). Similarity in the mean core area of each species isotopic niche was also indicated by the high SEA_B_ overlap between both species, with 63.82% for snubfin dolphins and 55.73% for humpback dolphins (Table 1, Figure 3). Mean distance to centroid (CD) was only marginally higher for humpback dolphins (1.22‰) than for snubfin dolphins (1.08‰) with 74.3% probability (Table 1). Mean nearest neighbor distance (MNND) and standard deviation of nearest neighbor distance (SDNND) were both higher in humpback dolphins (MNND = 0.85‰, SDNND = 0.48‰) than in snubfin dolphins (MNND = 0.33‰, SDNND = 0.30‰) (Table 1); however, bootstrapping showed low likelihood of these being different between species (MNND = 54.8% snubfin > humpback, SDNND = 62.1% humpback > snubfin).

**TABLE 1:** Isotopic niche metrics (including the six Layman metrics) for Australian snubfin and humpback dolphins

Metrics	Snubfin dolphin (*n* = 31)	Humpback dolphin (*n* = 23)	Probability
δ^15^N (mean ± 1 SD)	11.15 ± 1.02	11.33 ± 0.99	
δ^15^N range	4.4	3.7	
δ^15^N range_boot_	3.9	3.5	80.1% snubfin > humpback
δ^13^C (mean ± 1 SD)	−15.93 ± 0.85	−16.30 ± 1.14	
δ^13^C range	4.3	4.6	
δ^13^C range_boot_	3.4	4.2	84.8% humpback > snubfin
TA	12.07	10.66	
TA_boot_	8.22	9.91	72.0% humpback > snubfin
SEA	2.69	3.03	
SEA_C_	2.78	3.18	
SEA_B_	2.38	2.68	68.5% humpback > snubfin
SEA_B_ overlap	63.82	55.73	
CD	1.08	1.22	
CD_boot_	1.09	1.23	74.3% humpback > snubfin
MNND	0.37	0.52	
MNND_boot_	0.39	0.38	54.8% snubfin > humpback
SDNND	0.42	0.48	
SDNND_boot_	0.34	0.38	62.1% humpback > snubfin

Note

Isotopic means and ranges are given in ‰. Subscript “boot” indicates that the value (mean) has been generated via bootstrapping.

Abbreviations: CD, mean distance to centroid; MNND, mean nearest neighbor distance; SDNND, standard deviation of nearest neighbor distance; SEA, standard ellipse area; SEA_B_, Bayesian SEA; SEA_C_, standard ellipse area corrected for small sample size; TA, total area.

## 4 DISCUSSION

The interspecific similarities in isotope metrics found among snubfin and humpback dolphins in this study suggest that they feed at similar trophic levels, have substantial dietary overlap in isotopic niche space, and both rely on similar basal food resources. Despite similarities, snubfin dolphins were more likely to have a larger δ^15^N range and humpback dolphins a larger δ^13^C range, suggesting snubfin dolphins may feed on a slightly wider range of trophic levels and prey, while humpback dolphins may use a wider range of basal food resources. These findings are consistent with previous space use and stomach content analyses indicating that both species use similar habitats and feed on similar prey, but snubfins have a larger dietary breadth (Parra & Jedensjö, 2014) and humpback dolphins may use a greater diversity of habitats (Parra, 2006).

While stable isotope analysis can provide vital information into consumer–resource relationships, it is important to acknowledge that overlap in isotopic values of consumers does not necessarily indicate the same feeding habits or diet, as different prey species with similar isotopic values may produce similar δ^13^C and δ^15^N isotope values in their consumer’s tissue (Phillips et al., 2014; Santos‐Carvallo et al., 2015). Additionally, the resultant predator isotopic composition will also vary depending on the range of isotopic values and the relative proportions of ingested prey (Newsome et al., 2007; Phillips, 2001). Furthermore, foraging strategies often vary with geographic location, sex, and age in delphinids (Gannon & Waples, 2004; Rossman et al., 2015), and these differences together with spatial and temporal variation in basal resource availability can affect dolphin diet and hence their carbon and nitrogen isotopic values (Ansmann et al., 2015; Browning, Cockcroft, et al., 2014; Browning et al., 2014; Peters et al., 2020). In this study, sampling occurred over a small spatial and temporal scale, was restricted to adult dolphins, and did not include potential prey items to allow examination of these factors. Therefore, we recommend future isotopic studies include further sampling across different areas, seasons, dolphins of different age and sex, and a diverse range of potential prey species, to increase sample size, elucidate the influence of these factors on dolphin’s stable isotope values, and integrate with stable isotope mixing models to estimate the contribution of different prey sources. A more comprehensive approach including quantitative fatty acid signatures (Iverson et al., 2004) and compound‐specific stable isotope analyses (Twining et al., 2020) could also allow for more fine‐scale results. Despite these constraints, the results of this study are in line with our predictions and provide valuable insights into the trophic ecology of snubfin and humpback dolphins and a baseline for future studies.

Carbon isotope ratios in tissues of aquatic animals reflect the source of carbon at the base of the food chain and thus can be used to determine the habitat in which the predator has been feeding. (Kelly, 2000). In marine ecosystems, carbon isotope ratios tend to be more enriched in inshore/estuarine habitats than in offshore/pelagic environments (France, 1995; Fry & Sherr, 1989). Snubfin and humpback dolphins had δ^13^C values in the range expected for marine predators living and foraging in nearshore systems (Clementz & Koch, 2001; Yves & Keith, 2007). Stomach content analyses of stranded and shark‐net entangled dolphins revealed that both snubfin and humpback dolphins feed on a wide variety of fish and cephalopods associated with shallow coastal‐estuarine environments (Parra & Jedensjö, 2014). These feeding habits are in accordance with behavioral observations indicating that snubfin and humpback dolphins often feed in shallow, coastal‐estuarine habitats (Parra, 2006).

Interspecific differences in δ^15^N range are consistent with some degree of resource partitioning. The higher δ^15^N range observed in snubfin dolphins suggests they may feed on a slightly larger variety of prey resources than humpback dolphins. Stomach content analyses have shown that the main dietary difference between snubfin and humpback dolphins appears to be cephalopods, which were only found in large quantities in the stomachs of snubfin dolphins (Parra & Jedensjö, 2014). The cuttlefish and squid found in the stomachs of snubfin dolphins are abundant in shallow waters close to the coast (Jackson, 1991). In addition to cephalopods, snubfin dolphins also feed on schooling, bottom‐dwelling, and pelagic fishes (Parra & Jedensjö, 2014). Thus, it is likely that the higher δ^15^N range in snubfin dolphins reflects a greater variation in the trophic level of their diet due to the consumption of cephalopods, as well as fish.

In contrast, bootstrapping indicated humpback dolphins were more likely to have a larger δ^13^C range than snubfin dolphins, suggesting they may use a slightly wider diversity of habitats. Humpback dolphins are known to use a wide diversity of habitats associated with coastal waters, including dredged channels, inshore reefs, seagrass flats, and mangroves (Parra, 2006; Parra & Cagnazzi, 2016). Moreover, humpback dolphins have been sighted more than 50 km from the mainland coast in shallow shelf waters (i.e., <30 m deep) and near offshore islands off Queensland and Western Australia (Corkeron et al., 1997; Hanf et al., 2016; Parra et al., 2004; Raudino et al., 2018). Such sightings indicate that this species may use a wider range of different habitats, including intertidal areas around offshore islands. Alternatively, differences in δ^13^C range may reflect differences in basal resource availability across sampling locations in this study. The Capricorn Curtis region is characterized by a large inlet (Port Curtis), extended tidal‐dominated estuary (Fitzroy River), and a large shallow bay (Keppel Bay); whereas the Whitsundays region sampled here is characterized by coastal seagrass meadows, coral reefs, sandy embayments, coastal islands with fringing reefs, and sheltered lagoons. Further studies are needed to determine whether the estimated differences in δ^13^C range represent interspecific differences in the variability of trophic sources used by each dolphin species, or basal resource availability across sampling locations.

Despite the potential dietary differences indicated by δ^13^C and δ^15^N range, isotopic niche width metrics (TA and SEA_C_) were similar for both species and overlapped substantially (≥50%), suggesting high dietary overlap. These results are consistent with evidence from stomach content analysis which indicated that the fish prey in the diet of snubfin and humpback dolphins overlapped considerably. All fish taxa identified to genus level in humpback dolphin stomachs were also consumed by snubfin dolphins, and the most numerically important fish prey item in the stomach contents of each species (*Apogon* sp. for snubfin dolphins and *Pomadasys* sp. for humpback dolphins) was also consumed by the other (Parra & Jedensjö, 2014).

Ecological niche theory predicts that closely related species living in sympatry may compete for resources, unless there is resource partitioning through differences in dietary preferences, spatio‐temporal habitat use patterns, and/or feeding behavior (MacArthur, 1968; Pianka, 1981). The differences in δ^13^C and δ^15^N range between snubfin and humpback dolphins found in this study suggest there is potentially some level of dietary and habitat partitioning, with snubfin dolphins foraging over a wider diversity of prey resources than humpback dolphins, and humpback dolphins utilizing a larger variety of habitats. Such differences in prey selection and habitat utilization most likely help minimize interspecific competition and have been observed among other sympatric communities of delphinids (Ansmann et al., 2015; Browning, Cockcroft, et al., 2014; Giménez, Cañadas, et al., 2017; Kiszka et al., 2011).

At the same time, the substantial overlap (>50%) in the mean core area (40%) of each species’ isotopic niche space (SEA) suggests ecologically significant dietary overlap and potentially direct resource competition. Differences in dolphin feeding behavior may facilitate resource partitioning and reduce interspecific competition for shared prey resources such as fish. Humpback dolphins frequently forage behind trawlers, while snubfin dolphins have never been observed engaging in this behavior (Parra, 2006). Alternatively, coastal‐estuarine environments along the coast of Queensland are highly productive areas (Brodie et al., 2007), and as such, may provide abundant resources and promote snubfin and humpback dolphins’ coexistence despite a great deal of overlap in their diet.

Prey diversity and abundance are key factors promoting the coexistence of upper‐level predators as interspecific competition pressure tends to increase with decreasing prey availability and diversity (Holbrook & Schmitt, 1989; McArthur & Levins, 1967; Santos et al., 2019). Thus, anthropogenic activities impacting prey abundance and diversity (e.g., overfishing, pollution) can potentially affect the future coexistence of snubfin and humpback dolphins. As dolphins may play important roles in maintaining the structure and function of marine communities and ecosystems (Kiszka et al., 2022), cumulative anthropogenic pressures on their prey need to be considered when planning future multi‐species conservation.

## 5 CONCLUSIONS

Our results confirm and strengthen results from earlier studies that suggested there is a high overlap in the diet and habitat use of snubfin and humpback dolphins (Parra, 2006; Parra & Jedensjö, 2014). Despite the similarities, snubfin dolphins appear to feed on a wider range of trophic levels and prey, while humpback dolphins may feed in a larger variety of habitats; and such differences may be important factors promoting their coexistence. Analyses of dolphin and prey item isotope levels across different areas, seasons, dolphins of different age and sex, would help improve the interpretation of the isotopic results and elucidate the mechanisms of coexistence between these ecologically similar dolphin species.

## AUTHOR CONTRIBUTIONS

Guido J. Parra: Conceptualization (Lead); Data curation (Lead); Formal analysis (Lead); Funding acquisition (Supporting); Writing – original draft (Lead); Writing – review & editing (Lead). Zac Wojtkowiak Conceptualization (Lead); Data curation (Lead); Formal analysis (Lead); Writing – original draft (Lead); Writing – review & editing (Lead). Katharina Johanne Peters: Conceptualization (Supporting); Data curation (Supporting); Formal analysis (Supporting); Writing – original draft (Supporting); Writing – review & editing (Supporting). Daniele Cagnazzi: Conceptualization (Supporting); Data curation (Lead); Formal analysis (Supporting); Funding acquisition (Lead); Writing – original draft (Supporting); Writing – review & editing (Supporting).

## CONFLICT OF INTEREST

The authors declare no conflicts of interest in this study.

## Data Availability

Data associated with this manuscript have been deposited in Dryad at: https://doi.org/10.5061/dryad.95x69p8n0.
